# Improving reproducibility in animal research by splitting the study population into several ‘mini-experiments’

**DOI:** 10.1038/s41598-020-73503-4

**Published:** 2020-10-06

**Authors:** Vanessa Tabea von Kortzfleisch, Natasha A. Karp, Rupert Palme, Sylvia Kaiser, Norbert Sachser, S. Helene Richter

**Affiliations:** 1 Department of Behavioural Biology, University of Münster, Badestraße 13, Münster, Germany; 2 Otto Creutzfeldt Center for Cognitive and Behavioral Neuroscience, University of Münster, Münster, Germany; 3 Data Sciences and Quantitative Biology, Discovery Sciences, R&D, AstraZeneca, Cambridge, UK; 4 Department of Biomedical Sciences, University of Veterinary Medicine, Vienna, Austria

**Keywords:** Animal behaviour, Neuroscience, Emotion

## Abstract

In light of the hotly discussed ‘reproducibility crisis’, a rethinking of current methodologies appears essential. Implementing multi-laboratory designs has been shown to enhance the external validity and hence the reproducibility of findings from animal research. We here aimed at proposing a new experimental strategy that transfers this logic into a single-laboratory setting. We systematically introduced heterogeneity into our study population by splitting an experiment into several ‘mini-experiments’ spread over different time points a few weeks apart. We hypothesised to observe improved reproducibility in such a ‘mini-experiment’ design in comparison to a conventionally standardised design, according to which all animals are tested at one specific point in time. By comparing both designs across independent replicates, we could indeed show that the use of such a ‘mini-experiment’ design improved the reproducibility and accurate detection of exemplary treatment effects (behavioural and physiological differences between four mouse strains) in about half of all investigated strain comparisons. Thus, we successfully implemented and empirically validated an easy-to-handle strategy to tackle poor reproducibility in single-laboratory studies. Since other experiments within different life science disciplines share the main characteristics with the investigation reported here, these studies are likely to also benefit from this approach.

## Introduction

Concerns about the credibility of scientific results have become a major issue over the last years (e.g. Refs.^1–4^). This is aptly reflected by a recent survey of the Nature publishing group, which reported that over 90% of the interviewed researchers were convinced that science currently faces a ‘reproducibility crisis’^5^. Further evidence for this impression comes from several systematic replication studies in the field of biomedicine and psychology, where replication failed to an alarming extent^6–9^. Based on these studies, it was estimated that about 50–90% of the published findings are in fact irreproducible^10–12^. The reasons associated with poor reproducibility are numerous, ranging from fallacies in the experimental design and statistical analyses (e.g. p-hacking^13^ and HARKing^14^) to a lack of information in the published literature^10^.

Building on this discussion, specific guidelines, such as the TOP (Transparency and Openness Promotion)^15^, ARRIVE^16,17^, or PREPARE^18^ guidelines have been developed. Furthermore, to increase overall transparency in animal research and to counteract the problem of publication bias, the pre-registration of studies has been encouraged^19–22^. All of these attempts are promising strategies to improve the planning, analysis and reporting of studies^23^. However, as demonstrated by an already 20 years-old seminal study, the use of thoroughly planned and well-reported protocols does not automatically guarantee reproducibility^24^. In this study, three different laboratories simultaneously conducted the same animal experiment under highly standardised conditions. More precisely, they compared eight inbred mouse strains in a battery of six behavioural tests in each laboratory. Surprisingly, the three laboratories found remarkably different results, reflecting a typical example of what we call poor reproducibility (i.e. failure of obtaining the same results when replicating a study with a new study population). This observation was most likely due to the influence of many uncontrollable environmental background factors that affected the outcome of the experiment differently (e.g. noise^25^, microbiota^26^, or personnel^27^).

To embrace this kind of unavoidable variation within a single study and thereby to increase reproducibility, the idea to implement multi-laboratory study designs in animal research has been proposed recently^28^: In a simulation approach, 50 independent studies on the effect of therapeutic hypothermia on infarct volume in rodent models of stroke were used to compare the reproducibility of treatment effects between multi-laboratory and single-laboratory designs. And indeed, by re-analysing these data, the authors demonstrated that multi-laboratory studies produced much more consistent results than single-laboratory studies^28^.

However, multi-laboratory studies are logistically challenging and are not yet suitable to replace the broad mass of single-laboratory studies. Therefore, solutions are urgently needed to tackle the problem of poor reproducibility at the level of single-laboratory studies. Against this background, the overall idea of the present study was to design an experimental strategy that transfers the multi-laboratory logic into a single-laboratory setting, and at the same time offers a high degree of practical relevance.

The essential element of the above described multi-laboratory approach is the inclusion of heterogeneity within the study population. In particular, by mimicking the inevitably existing between-laboratory variation within one study, the representativeness of the study population is enhanced. For instance, actively integrating background factors that are usually not in the focus of the study such as changing personnel or the temperature, is supposed to render the results more generalisable. This way, the external validity is increased, leading to better reproducibility of research outcomes across independent replicate experiments^29,30^. In line with this assumption, there is accumulating theoretical and empirical evidence that systematic and controlled heterogenisation of experimental conditions within single laboratories also increases the reproducibility of research outcomes in comparison to rigorously standardised experiments^31–34^. However, an effective and at the same time easy-to-apply heterogenisation strategy is still missing^29,33,35^.

**Figure 1:**
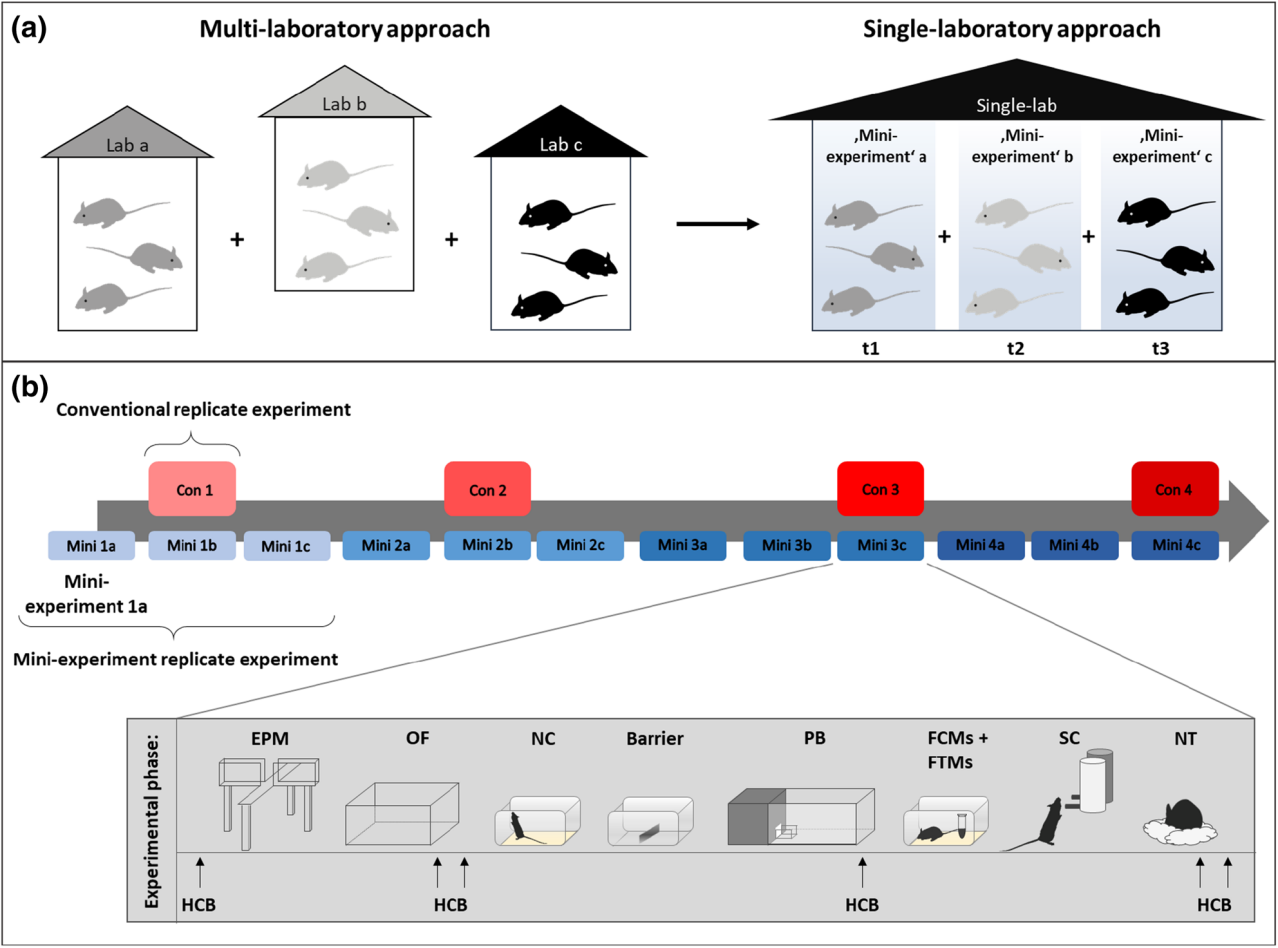
Concept of the study. (**a**) Transfer of the multi-laboratory approach into a single-laboratory situation. In the multi-laboratory situation, the integration of different laboratory environments in one study results in a heterogenous study population. In the single-laboratory approach, the animals are tested in the same laboratory, but in different mini-experiments spread over three time points (t1–t3). Between mini-experiments, uncontrollable factors of the laboratory environment may vary in the same way as they may vary between laboratories in the multi-laboratory approach. Thereby, the heterogeneity of the study population is enhanced, resembling the logic of the multi-laboratory approach. (**b**) Overview of the study design: Strain differences were repeatedly investigated in four independent replicate experiments in both a conventional (Con, red) and a mini-experiment design (Mini, blue). In the conventional design, all animals of one replicate experiment (e.g. Con 1) were tested at one specific point in time. In the mini-experiment design, by contrast, one replicate experiment (e.g. Mini 1) was split in three mini-experiments (Mini 1a, Mini 1b, and Mini 1c), all organised in the same way. Please note, whenever mice of a conventional replicate experiment were tested, also one mini-experiment of the corresponding mini-experiment replicate experiment was conducted to control for potential time-point specific background effects. Experimental phase: *EPM* Elevated Plus Maze, *OF* Open Field test, *NC* Novel Cage test, *Barrier* Barrier test, *PB* Puzzle Box test, *FCMs + FTMs* Collection of faecal samples for assessment of corticosterone and testosterone metabolites, *SC* Sucrose Consumption test, *NT* Nest test, *HCB* Home cage behaviour. In addition, body weights were taken.

For the successful implementation of such a strategy, the following two requirements have to be met: First of all, considering the logic of the multi-laboratory approach, factors need to be identified that inevitably vary between experiments as they would vary between laboratories in a multi-laboratory setting and are not part of the study question. A promising and repeatedly highlighted candidate in this respect is the time of testing throughout the year (referred to as ‘batch’ for example by Refs.^29,36,37^). This factor has not only been shown to substantially influence the phenotype of mice tested in the same laboratory^38,39^, but can also be regarded as sort of an ‘umbrella factor’ for plenty of uncontrollable varying known and unknown background factors (e.g. changing personnel, noise, temperature, etc.). By covering a diverse spectrum of background heterogeneity, variation of this factor thus automatically enhances the representativeness of the study population as the variation of the laboratory environment does in the multi-laboratory approach. Second, a successful implementation critically relies on the feasibility of the approach and its potential to introduce the necessary variation in a systematic and controlled way. Again, the time of testing throughout the year appears a promising factor: It can be easily varied in a systematic and controlled way, and the implementation appears feasible as it simply implies to collect data over time. Against this background, we here propose and validate an experimental strategy that builds on systematically varying the time of testing by splitting an experiment into several independent ‘mini-experiments’ conducted at different time points throughout the year (referred to as ‘mini-experiment’ design in the following, see Fig. 1). In light of the above presented conceptual framework, we hypothesise to observe improved reproducibility of research findings in such a mini-experiment design in comparison to a conventionally standardised design, according to which all animals are tested at one specific point in time (referred to as conventional design in the following, see Fig. 1).

## Results

In experimental animal research, many studies examine the role of specific genes in the modulation of the phenotype and therefore rely on the phenotypic characterisation of genetically modified animals. To mimic such an experiment with a typical ‘treatment under investigation’ (i.e. different genotypes), behavioural and physiological differences between mouse strains were investigated in tests commonly used in such phenotyping studies^40^. In detail, male mice of the three inbred mouse strains C57BL/6J, DBA/2N and BALB/cN, and the F1 hybrid strain B6D2F1N were tested in a battery of well-established behavioural and physiological paradigms. The battery included the examination of exploratory and anxiety-like behaviours, hedonic states, cognitive abilities, nest building, spontaneous home cage behaviour, body weight changes, and hormonal profiles (i.e. corticosterone and testosterone metabolite concentrations, for details see “Experimental phase” in the “Methods” section). Previous studies have shown that the selected strains differ, for instance, in their anxiety-like and learning behaviour (C57BL/6 vs BALB/c and DBA/2)^24,41–43^, but not in their exploratory locomotion (C57BL/6 vs BALB/c)^24,41^.

The above described examination was done in two experimental designs, namely a mini-experiment design and a conventional design, which were then compared with respect to their effectiveness in terms of reproducibility. More precisely, to examine the reproducibility of the strain differences in both designs, a total of four conventional and four mini-experiment replicate experiments were conducted successively over a time period of 1.5 years (conventional design: Con 1–Con 4 and mini-experiment design: Mini 1–Mini 4, Fig. 1b). Within each conventional replicate experiment and each mini-experiment replicate experiment, a sample size of 9 mice per strain were tested. In the mini-experiment design, however, one replicate experiment was split in three mini-experiments (Mini 1a, Mini 1b, Mini 1c, Fig. 1b), each comprising a reduced number of animals at one specific point in time (i.e. 3 per strain). Thus, whereas all mice of one conventional replicate experiment (i.e. 9 per strain) were delivered and tested at one specific point in time, in the mini-experiment design, they were delivered and tested in three mini-experiments that were conducted at different time points throughout the year (i.e. 3 mice per strain per mini-experiment). Consequently, factors, such as the age of the animals at delivery or the age of the animals at testing were the same for all mini-experiments and the conventional replicate experiments. Furthermore, concerning factors, such as for example temperature, personnel, and type of bedding, conditions were kept as constant as possible within mini-experiments, whereas between mini-experiments conditions were allowed to vary (for details see Supplementary Table S1). This approach was taken to reflect the usual fluctuations in conditions between independent studies.

Both designs were organised according to a randomised block design. In the mini-experiment design, one ‘block’ corresponded to one mini-experiment and in the conventional design, one ‘block’ subsumed cages of mice positioned within the same rack and tested consecutively in the battery of tests (for details see Supplementary Fig. S1).

Reproducibility was compared between the conventional and the mini-experiment design on the basis of behavioural and physiological differences between the four mouse strains (yielding 6 strain comparisons in total). In particular, two different approaches were taken to tackle the issue of reproducibility from a statistical perspective: (I) Consistency of the strain effects across replicate experiments and (II) Estimation of how often and how accurately the replicate experiments predict the overall effect.

### (I) Consistency of the strain effects across replicate experiments

The consistency of the strain effect across replicate experiments is statistically reflected in the interaction term of the strain effect with the replicate experiments (‘strain-by-replicate experiment’-interaction). To assess this interaction term for all outcome measures of each strain comparison, we applied a univariate linear mixed model (LMM; for details please see the “Methods” section) to both designs. Usually, the contribution of a fixed effect to a model is determined by examining the F-values as they return the relative variance that is explained by the term against the total variance of the data. As this LMM is a random effect mixed model accounting for the structure in the data and the F-values cannot be assessed for a random effect (i.e. ‘strain-by-replicate-experiment’-interaction), we used the p-value of the interaction term as a proxy for the F-test (for details see “Methods” section; analysis adapted from Ref.^32^, and see also Ref.^44^). A higher p-value of the interaction term indicates less impact of the replicate experiments on the consistency of the strain effect (i.e. better reproducibility). Therefore, p-values of the interaction term for 16 representative outcome measures for each strain comparison were compared between the designs using a one-tailed Wilcoxon signed-rank test (the selected measures covered all paradigms used, for details please see the “Methods” section).

**Figure 2:**
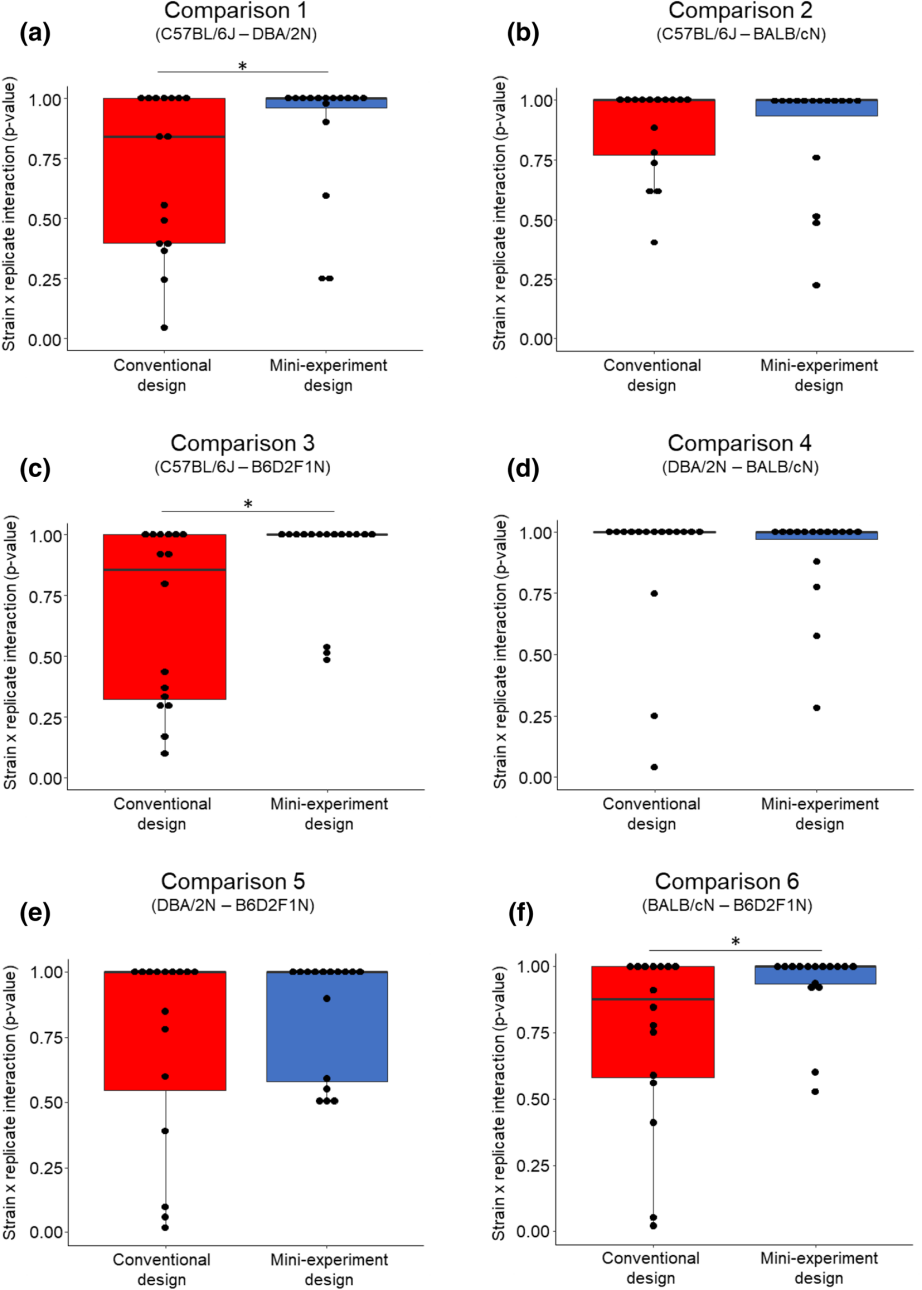
Consistency of the strain effect across replicate experiments for all strain comparisons, respectively, of both the conventional (red) and the mini-experiment (blue) design. Shown are p-values of the ‘strain-by-replicate experiment’-interaction term across all 16 outcome measures. Data are presented as boxplots showing medians, 25% and 75% percentiles, and 5% and 95% percentiles. Black dots represent single p-values for each outcome measure in both designs. Statistics: Wilcoxon signed-rank test (paired, one-tailed, n = 16), *p ≤ 0.05.

The p-values of the ‘strain-by-replicate experiment’-interaction term were significantly higher in the mini-experiment than in the conventional design in 3 out of 6 strain comparisons, demonstrating improved reproducibility among replicate experiments in the mini-experiment design in half of all investigated exemplary treatment effects (Fig. 2a, c, f; Wilcoxon signed-rank test (paired, one-tailed, n = 16): Comparison 1 ‘C57BL/6J–DBA/2N’: V = 21, p-value = 0.047; Comparison 3 ‘C57BL/6J–B6D2F1N’: V = 11, p-value = 0.009; Comparison 6 ‘BALB/cN–B6D2F1N’: V = 5, p-value = 0.003). For the remaining three strain comparisons, however, no significant differences were found (Fig. 2b, d, e; Wilcoxon signed-rank test (paired, one-tailed, n = 16): Comparison 2 ‘C57BL/6J–BALB/cN’: V = 35.5, p-value = 0.429; Comparison 4 ‘DBA/2N–BALB/cN’: V = 59.5, p-value = 0.342; Comparison 5 ‘DBA/2N–B6D2F1N’: V = 48, p-value = 0.257). Here, both designs were characterised by a high median p-value of the interaction term, reflecting a rather good reproducibility independent of the experimental design (see Fig. 2b, d, e).

**Figure 3:**
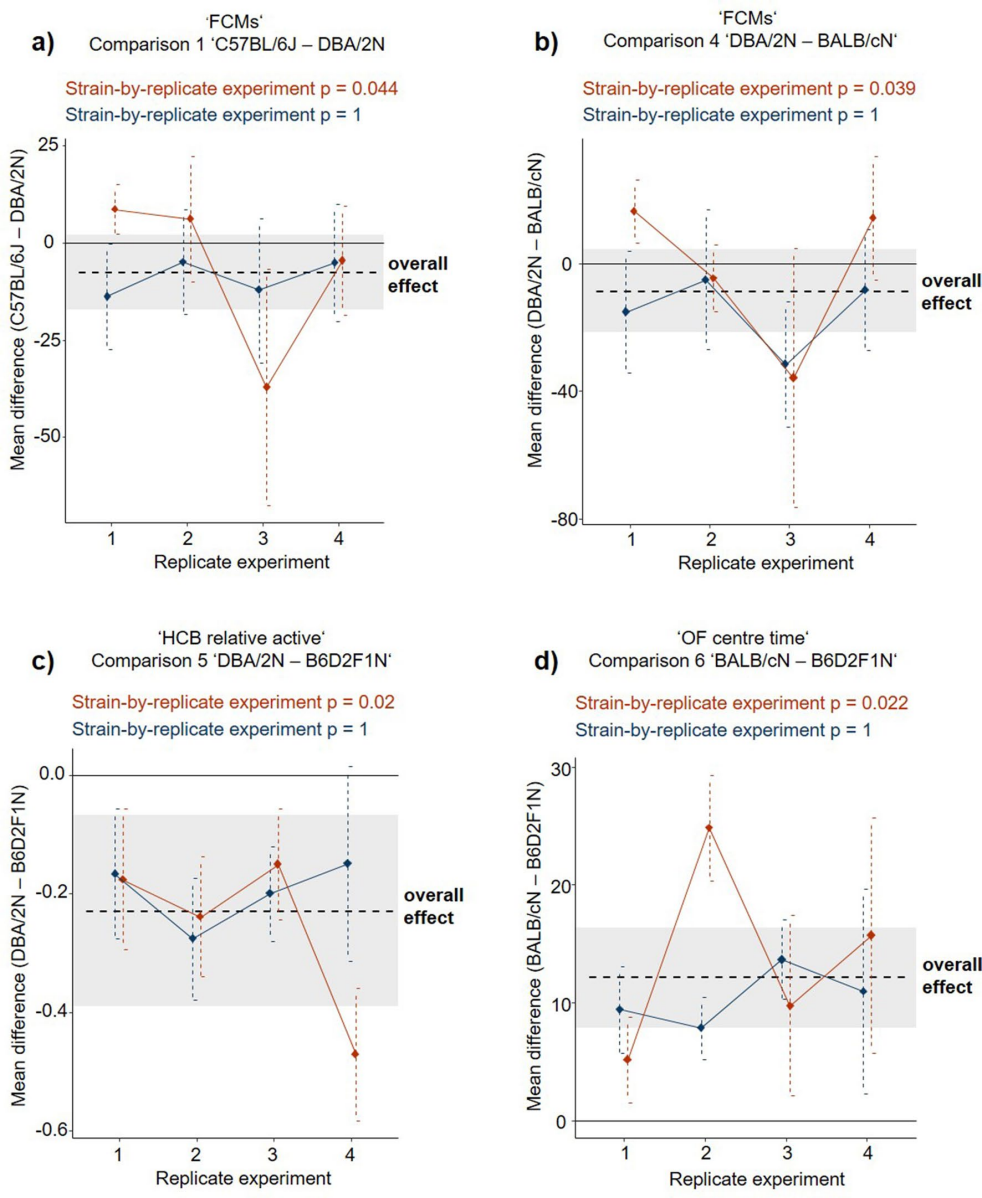
Variation of mean strain differences across the four replicate experiments in the conventional (red) and the mini-experiment design (blue). Shown are the four outcome measures, which yielded a significant ‘strain-by-replicate experiment’-interaction effect (i.e. impaired reproducibility) in one of the experimental designs. Notably, we found significant ‘strain-by-replicate experiment’-interaction effects only in the conventional design. Shown are mean strain differences in (**a**) ‘faecal corticosterone metabolite concentrations’ in comparison 1 ‘C57BL/6J–DBA/2N’. (**b**) ‘faecal corticosterone metabolite concentrations’ in comparison 4 ‘DBA/2N–BALB/cN’. (**c**) ‘proportion of 'active' home cage behaviour observations’ in comparison 5 ‘DBA/2N–B6D2F1N’ and (**d**) ‘Open Field centre time’ in comparison 6 ‘BALB/cN–B6D2F1N’. The black dashed line and the shaded area indicate the overall mean strain difference of this parameter and its corresponding 95% confidence interval (CI_95_). The black solid line reflects a null effect. Dots and vertical dashed lines reflect the mean strain differences and corresponding CI_95_ of the four replicate experiments in each design.

Looking at single outcome measures for all strain comparisons of both designs, we found four significant strain-by-replicate experiment-interactions, but only in the conventional design (a p-value of < 0.05 was detected in 4 out of 96 interactions, Fig. 3 and Supplementary Tables S2–S7, for a graphical overview of all single outcome measures please see Supplementary Figs. S2–S7). Such a significant interaction term highlights a strain effect that is not consistent across replicate experiments and thus indicates hampered reproducibility. Interestingly, as depicted in the forest plots in Fig. 3, some conventional replicate experiments predicted strain effects in even opposite directions with non-overlapping confidence intervals (Fig. 3a, b; conventional replicate experiment 1 versus 3). Please note that these events of hampered reproducibility rely on a descriptive comparison of the two designs and do not allow for inferential conclusions.

### (II) Estimation of how often and how accurately the replicate experiments predict the overall effect

In the second analysis, reproducibility was investigated by comparing the performance of the designs to predict the overall effect size (see Ref.^28^). The overall effect size (i.e. the mean strain difference) and corresponding 95% confidence intervals (CI_95_) were estimated from the data of replicate experiments of both designs by conducting a random-effect meta-analysis (see “Methods” for details). This was completed for each outcome measure and each strain comparison. Within each replicate experiment of both designs, individual effect sizes and CI_95_ were calculated (Fig. 3). Finally, the individual estimated effects of each replicate experiment were compared to the overall effects using the following two measurements: First, the coverage probability (Pc) was assessed by counting how often the CI_95_ of the replicate experiments in each experimental design covered the overall effect size (replicate experiments marked by ♣ in Supplementary Fig. S8). Second, the proportion of accurate results (Pa) was calculated by counting those replicate experiments that predicted the overall effect size accurately with respect to their statistical significance (replicate experiments marked by ♦ in Supplementary Fig. S8). Subsequently, Pc and Pa ratios of all 16 outcome measures were compared between both experimental designs using a one-tailed Wilcoxon signed-rank test.

**Figure 4:**
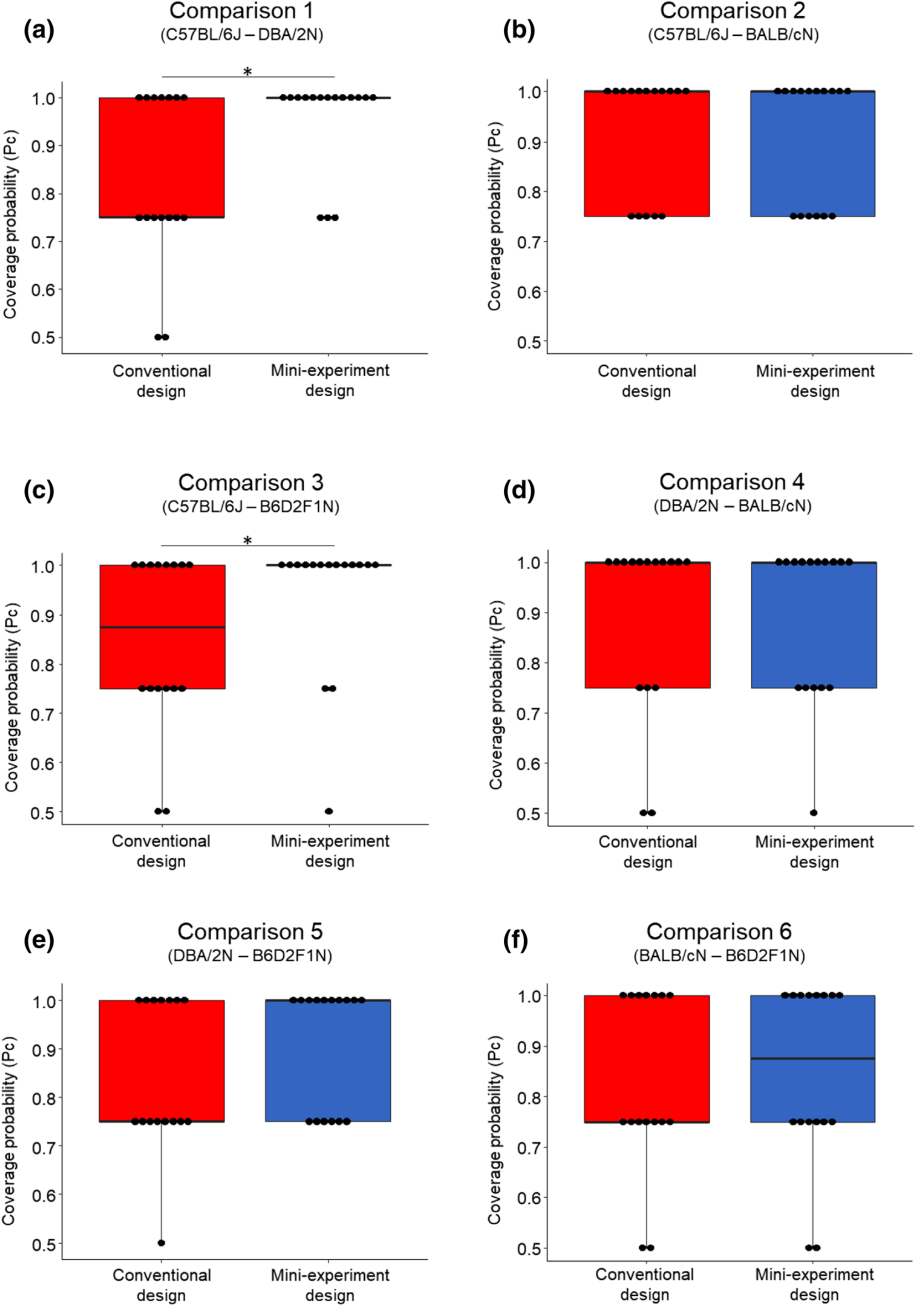
Comparison of the coverage probability (Pc) between both experimental designs. Pc of all 16 outcome measures in the conventional (red) and mini-experiment design (blue) for all six strain comparisons, respectively. Data are presented as boxplots showing medians, 25% and 75% percentiles, and 5% and 95% percentiles. Black dots represent single Pc values for each outcome measure in both designs. Statistics: Wilcoxon signed-rank test (paired, one-tailed, n = 16), *p ≤ 0.05.

**Figure 5:**
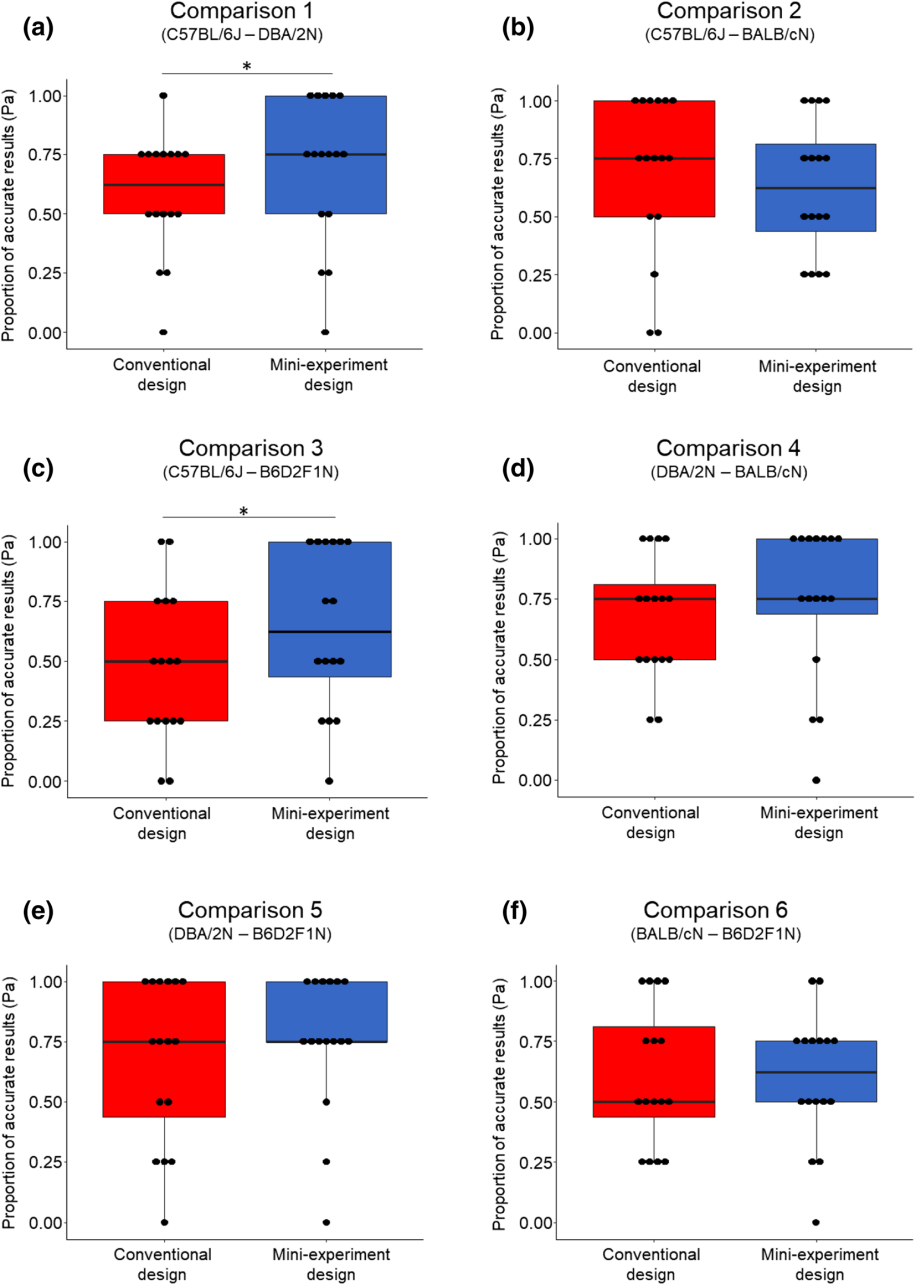
Comparison of the proportion of accurate results (Pa) between both experimental designs. Pa of all 16 outcome measures in the conventional (red) and mini-experiment design (blue) for all six strain comparisons, respectively. Data are presented as boxplots showing medians, 25% and 75% percentiles, and 5% and 95% percentiles. Black dots represent single Pa values for each outcome measure in both designs. Statistics: Wilcoxon signed-rank test (paired, one-tailed, n = 16), *p ≤ 0.05.

In line with our previous findings, the replicate experiments in the mini-experiment design covered the overall effect significantly more often than the replicate experiments in the conventional design (higher Pc ratio) for two out of six comparisons (Fig. 4a, c; Wilcoxon signed-rank test (paired, one-tailed, n = 16): Comparison 1 ‘C57BL/6J–DBA/2N’: V = 4.5, p-value = 0.012; Comparison 3 ‘C57BL/6J–B6D2F1N’: V = 3.5, p-value = 0.035). Furthermore, focusing on these comparisons, the Pa was significantly higher in the mini-experiment than in the conventional design, again demonstrating better reproducibility in the mini-experiment design (Fig. 5a, c; Wilcoxon signed-rank test (paired, one-tailed, n = 16): Comparison 1 ‘C57BL/6J–DBA/2N’: V = 10, p-value = 0.03; Comparison 3 ‘C57BL/6J–B6D2F1N’: V = 4.5, p-value = 0.008). With respect to the remaining four strain comparisons, no significant differences in the Pc and the Pa between both designs could be found (Figs. 4b,d–f, 5b,d–f); Wilcoxon signed-rank test (paired, one-tailed, n = 16): Pc: Comparison 2 ‘C57BL/6J–BALB/cN’: V = 4, p-value = 0.386; Comparison 4 ‘DBA/2N–BALB/cN’: V = 14, p-value = 0.5; Comparison 5 ‘DBA/2N–B6D2F1N’: V = 2.5, p-value = 0.102; Comparison 6 ‘BALB/cN–B6D2F1N’: V = 12, p-value = 0.388; Pa: Comparison 2 ‘C57BL/6J–BALB/cN’: V = 42.5, p-value = 0.201; Comparison 4 ‘DBA/2N–BALB/cN’: V = 12, p-value = 0.204; Comparison 5 ‘DBA/2N–B6D2F1N’: V = 10.5, p-value = 0.153; Comparison 6 ‘BALB/cN–B6D2F1N’: V = 20, p-value = 0.411).

## Discussion

In light of the extensively discussed reproducibility crisis, introducing heterogeneity in the study population by implementing multi-laboratory study designs has been shown to enhance the external validity and hence the reproducibility of findings from animal research^28^. We aimed at transferring this logic to single laboratories to introduce likewise heterogeneity in the study population in a systematic and controlled way. In detail, an experimental strategy using independent mini-experiments spread over three different time points just a few weeks apart was applied and empirically tested regarding its reproducibility of the results in comparison to a conventional standardised design. Indeed, we observed improved reproducibility of the results from the mini-experiment design across independent replicates in about half of all investigated treatment effects. More precisely, improved reproducibility was reflected by a significantly lower between-replicate experiment variation in three out of six strain comparisons. Furthermore, replicate experiments in the mini-experiment design predicted the overall found effect size significantly more often and more accurately in two out of six strain comparisons. With respect to the remaining strain comparisons, in both experimental designs a rather good reproducibility of strain differences was observed. For instance, the results of the comparison between the two inbred strains C57BL/6 and BALB/c turned out to be very robust in our study in both designs. As previous studies found fluctuating results regarding exactly this strain difference^24,41^, it is likely that no further improvement by the mini-experiment design was detectable due to a ceiling effect (i.e. nearly no variation of the strain effect across the replicate experiments was detectable in both experimental designs).

Besides these overall effects, we also investigated outcome measures separately and detected events of severely hampered reproducibility. Remarkably, these problems exclusively occurred in the conventional design with strain differences pointing in opposite directions. Similar to the landmark study of Crabbe et al.^24^, these observations represent again examples of irreproducibility that are not due to a lack of planning or reporting standards, but to the high idiosyncrasy of results from rigorously standardised experiments. This problem has been discussed to be particularly acute in animal research, as animals and other living organisms are highly responsive in their phenotype to environmental changes. This phenotypic plasticity^45^ can result in an altered response (i.e. reaction norm^46^) towards a treatment effect depending on the environmental conditions of an experiment^30^. Therefore, even subtle and inevitable changing experimental conditions may lead to completely contradicting conclusions about the treatment under investigation (in our case strain differences). This might even occur when an experiment is replicated in the same laboratory, but for example the animals are purchased by another vendor^47^, tested by another experimenter^48^ or at another time of the day^34^. In line with these examples, 50% of interviewed scientists stated they have experienced failures in replicating their own experiments^5^. This prevalence of idiosyncratic findings in the literature highlights the need for a strategy that decreases the risk of finding results which are only valid under narrowly defined conditions (e.g. one specific experimenter) and therefore not of biological interest.

Introducing heterogeneity by means of a mini-experiment design efficiently reduced the risk to obtain replicate-specific and hence irreproducible findings in the present study. This is in line with accumulating theoretical^28,31,34^ and empirical^32,33,49^ evidence that systematic heterogeneity in the study population plays an important role for avoiding spurious findings. In contrast to previously suggested heterogenisation strategies, however, the proposed mini-experiment design does not require the variation of specific, a priori defined experimental factors, such as for example the age of the experimental subjects or the housing conditions^32,33^. Instead, this strategy uses the heterogenisation factor ‘time point throughout the year’ which includes known and unknown background factors that uncontrollably vary over time and hence differ automatically between mini-experiments. As a consequence, the mini-experiment design utilises in particular those background factors, which are typically neither controlled for nor systematically investigated as factors of interest (e.g. noise, changing personnel, season). Please note that the extent to which these background factors vary might differ from study to study. Since the efficiency of the here proposed heterogenisation strategy is linked to the variation of these known and unknown background factors over time, studies can benefit to different extents. Similar to the logic of the multi-laboratory approach, the mini-experiment design covers the inevitably existing between-replicate variation within one experiment (i.e. each mini-experiment in the mini-experiment design is analogous to one laboratory in the multi-laboratory design) to make the results more robust across the variation that we usually observe between independent studies. Whether the mini-experiment design provides a solution to improve the reproducibility of results not only across independent replicates in the same laboratory, but also across different laboratories, however, needs further validation in a real-life, multi-laboratory situation.

Furthermore, the mini-experiment design stands out by its practical feasibility. Although the time span to conduct a study is expanded by spreading the experiment across time, it provides several practical benefits in return. First, in contrast to a conventional experiment, in each mini-experiment a reduced number of animals is tested per time point. This is particularly beneficial for studies with genetically modified animals. Transgenic or knockout mouse models, for example, are often characterised by small breeding rates, so that not all experimental subjects are born at one specific time point, but over the course of several weeks (e.g. phenotyping studies of genetically modified mice^50^). As a mini-experiment design relies on testing animals in small successive batches, it provides a systematic solution for ‘collecting’ data of these animals over time. Second, time-demanding experiments, such as for example complex learning tasks (e.g. Ref.^51^), may benefit from a mini-experiment design, because the workload per day is drastically reduced.

Despite these conceptual and practical advantages of utilising more heterogeneous study populations in animal research, rigorously standardised experimental conditions are still accepted to be the gold-standard, also for ethical reasons. By reducing variation in the study population, standardisation is assumed to increase test sensitivity and thereby to reduce the number of animals needed to detect an effect^52^. Following this logic, one could assume that more heterogeneous study populations would require more animals to detect a significant effect. Indeed, this argument may hold true for the introduction of uncontrolled variation in the data (i.e. noisy data)^53^. However, the mini-experiment design introduces variation in a systematic and controlled way by using a randomised block design where each mini-experiment corresponds to one ‘block’. In fact, such designs have been suggested to be particularly powerful and of high external validity as long as the random blocking factor is considered in the statistical analysis^54^. In line with this, our study demonstrate that the mini-experiment design led to more reproducible results compared to the conventionally standardised design in about half of all investigated treatment effects, even though the total number of animals used was identical. Moreover, the mini-experiment design led to a higher proportion of accurate results (Pa) and hence provided more accurate conclusions than the conventional design in one third of the investigated treatment effects. These findings highlight that the above presented argument of increased test sensitivity through rigorous standardisation might come at the cost of obtaining less accurate and hence idiosyncratic results (see also Ref.^28^). In line with this, a comprehensive simulation study found that testing in multiple batches (i.e. mini-experiments) provides more confidence in the results than testing in one ‘big’ batch^55^. Therefore, arguing from a 3R-perspective^56^ (i.e. Replace, Reduce, Refine), a mini-experiment design increases the informative value of an experiment without requiring higher sample sizes than traditional designs. It thus contributes to both the ‘Refinement’ of experiments by enhancing the external validity and reproducibility of findings, and the ‘Reduction’ of animal numbers by decreasing the need for obsolete replication studies^28,57^.

In conclusion, we here proposed and empirically validated a novel mini-experiment design, which may serve as an effective and at the same time easy-to-apply heterogenisation strategy for single-laboratory studies. We believe that particularly those studies may benefit from a mini-experiment design that can be influenced by seasonal changes in the experimental background, as this approach fosters greater generalisability and thereby helps to avoid idiosyncratic (i.e. time-specific) results. Although tested by the example of behavioural and physiological mouse strain differences, benefits of applying such a design may not be limited to the field of animal research. In this respect, a study investigating grass-legume mixtures in a simple microcosm experiment has already shown that the introduction of heterogeneity on the basis of genetic and environmental variation in the experimental design can also enhance the reproducibility of ecological studies^49^. Therefore, using a mini-experiment design to include heterogeneity systematically in the study population may also benefit the reproducibility in other research branches of the life sciences.

## Methods

### Animals and housing conditions

For this study, male mice of three inbred (C57BL/6J, DBA/2N and BALB/cN) and one F1 hybrid strain (B6D2F1N) were provided by one supplier (Charles River Laboratories). All mice arrived at an age of about 4 weeks (PND 28). Upon arrival, the animals were housed in same strain groups of three mice per cage until PND 65 ± 2. Thereafter, mice were transferred to single housing conditions to avoid any kind of severe inter-male aggression within group housing conditions (for ongoing discussions about how to house male mice please see Refs.^58,59^). In both phases (group and single housing conditions), the animals were conventionally housed in enriched Makrolon type III cages (38 cm × 22 cm × 15 cm) filled with bedding material (the standard bedding material used in our facility changed over the course of this study from Allspan, Höveler GmbH & Co.KG, Langenfeld, Germany to Tierwohl, J. Reckhorn GmbH & Co.KG, Rosenberg, Germany) and equipped with a tissue paper as nesting material, a red transparent plastic house (Mouse House, Tecniplast Deutschland GmbH, Hohenpeißenberg, Germany) and a wooden stick. Food pellets (Altromin 1324, Altromin Spezialfutter GmbH & Co. KG, Lage, Germany) and tap water were provided ad libitum. Health monitoring took place and cages were cleaned weekly in the group housing phase and fortnightly afterwards. Enrichment was replaced fortnightly in both phases. Housing rooms were maintained at a 12/12 h light/dark cycle with lights off at 9:00 a.m., a temperature of about 22 °C and a relative humidity of about 50%.

### Ethics statement

All procedures complied with the regulations covering animal experimentation within Germany (Animal Welfare Act) and the EU (European Communities Council DIRECTIVE 2010/63/EU) and were approved by the local (Gesundheits-und Veterinäramt Münster, Nordrhein-Westfalen) and federal authorities (Landesamt für Natur, Umwelt und Verbraucherschutz Nordrhein-Westfalen “LANUV NRW”, reference number: 84-02.04.2015.A245).

### Experimental phase

To examine differences between the four mouse strains, all mice of all replicate experiments were subjected to the same experimental testing procedures, including the investigation of exploratory and anxiety-like behaviours, hedonic states, cognitive abilities, nest building and spontaneous home cage behaviour, bodyweight changes, and hormonal profiles. This was done to reflect data from both behavioural and physiological measurements. Behavioural paradigms were chosen in accordance to established protocols for the phenotypic characterisation of mice in animal research^40^. In each replicate experiment, 9 mice per strain (n = 9) were tested, except for two replicate experiments in the mini-experiment design (Mini 1, Mini 2). Here the sample size of one strain (‘B6D2F1N’) was reduced to n = 8, due to the death of 2 mice before the start of the experimental phase.

The experimental phase started for all animals on postnatal day (PND) 73 ± 1 and lasted for three weeks. Spontaneous home cage behaviour (HCB) was observed on six days between PND 73 ± 1 and PND 93 ± 1 and faecal samples to determine corticosterone and testosterone metabolite concentrations were collected on PND 87. Additionally, the bodyweight of the animals on PND 65 ± 2 as well as the weight gain over the test phase, from PND 73 ± 1 to PND 93 ± 1, was measured. Throughout the experimental phase, the following tests were conducted during the active phase in the same order for all animals: Elevated Plus Maze (EPM) on PND 77 ± 2, Open Field test (OF) on PND 79 ± 2, Novel Cage test (NC) on PND 83, Barrier test on PND 84, Puzzle Box test (PB) on PND 85 ± 1, Sucrose Consumption test (SC) starting on PND 87 and Nest test (NT) starting on PND 91 (see Fig. 1).

The EPM, OF and PB were conducted under dim light conditions in a testing room separated from the housing room. The NC, Barrier, SC and NT were conducted in the housing room under red light conditions. In all paradigms, the order of the mice was pseudo-randomised following two rules. First, always four mice, one out of each strain, were tested consecutively. Second, the order of these four mice was randomised with respect to the strains. All mice, regardless of the replicate experiment, were tested by the same experienced experimenter in all test procedures, except for the Nest test. In the latter, two experimenters scored the behavioural data of all mice simultaneously, regardless of the replicate experiment (for detail see the section “Nest test” below).

As strains had different fur colours (C57BL/6J and B6D2F1N mice: black, DBA/2N mice: brown, BALB/cN mice: white), blinding was not possible at the level of the exemplary treatment groups. Therefore, it cannot be excluded that the experimenter might have unconsciously influenced the animal’s behaviour and thus, the observed strain differences. However, we were not interested in the actual strain differences, but instead investigated the reproducibility of these strain comparisons. The reproducibility of strain differences across replicate experiments, however, was unlikely to be influenced by the presence or absence of blinding procedures at the level of strains. Importantly, the crucial level of blinding in this study was based on the experimental design. For this reason, the experimenters were blind with respect to the allocation of the mice to the experimental design (conventional or mini-experiment), whenever experiments in both designs were conducted at the same time. This involved four out of twelve mini-experiments (one from each replicate experiment) and all four conventional replicate experiments. By this, the experimenters were not aware which animals were tested in the conventional standardised design at all.

In the following, details on the test procedures during the experimental phase are given:

#### Elevated Plus Maze test

The EPM was conducted to examine exploratory locomotion and anxiety-like behaviour of the animals^60^. The apparatus was elevated 50 cm above the ground and was composed of two opposing open (30 cm × 5 cm) and two opposing closed arms (30 cm × 5 cm) which were connected via a central square (5 cm × 5 cm). The closed arms were surrounded by 20 cm high walls. The EPM was illuminated from above (25 lx in the centre square). After spending 1 min in an empty box protected from light, the mouse was placed on the central platform facing a closed arm and was allowed to freely explore the apparatus for 5 min. During that time, the animal was recorded by a webcam (Webcam Pro 9000, Logitech) in the absence of the experimenter. Outcome measures taken were the relative amount of entries into and the relative time spent in the open arms [i.e. open arm entries or time/(open arm + closed arm entries or time)]. In addition, the number of protected head dips (‘mouse lowers its head over the side of an open arm with its ears protruding over the edge, while at least one paw remains in the closed segment or central platform’; cf.^33^) was calculated relative to the total amount of head dips shown.

#### Open Field test

Similar to the EPM, the OF is a paradigm to assess the exploratory locomotion and anxiety-like behaviour of mice^61^. The apparatus consisted of a square arena (80 cm × 80 cm) surrounded by 40 cm high walls and illuminated from above (35 lx). A centre zone was defined as a 40 cm × 40 cm square area located in 20 cm distance from all walls. After spending 1 min in an empty box protected from light, the animal was placed in one corner of the OF facing the wall and was allowed to freely explore the arena for 5 min. During that time, the animal was recorded by a webcam (Webcam Pro 9000, Logitech) in the absence of the experimenter. The number of entries into the centre zone, the distance travelled and the time spent in the centre zone as well as the total distance travelled in the OF was automatically analysed by the video tracking software ANY-maze (Version 4.99 or 5.31, Stoelting Co.). In addition, the number of faecal boli in the OF was counted.

#### Novel Cage test

In the NC, exploratory locomotion was observed in a new environment by resembling a cage cleaning routine^62^. A new empty Makrolon type III cage (standard housing cage) was filled with 1 L of bedding material. Mice were placed into this new housing cage and the frequency of ‘rearing’ (‘a mouse raises itself on its hindpaws and stretches its snout into the air’) was recorded as a measure of vertical exploration by the experimenter for a duration of 5 min.

#### Barrier test

The Barrier test apparatus consisted of an empty Makrolon cage type III (standard housing cage), which was divided in half by a 3 cm high acrylic, transparent barrier. The mice were placed in one half facing the wall and the latency to climb over the barrier was measured to assess the exploratory locomotion^63^. The maximum duration of the test was set to 5 min.

#### Puzzle Box test

The PB test is a simple task to assess learning and problem-solving ability in mice (based on Ref.^64^). The rectangular shaped apparatus had the dimensions 75 cm × 28 cm × 25 cm and was divided in a light (60 cm × 28 cm) and a dark (15 cm × 28 cm) compartment illuminated from above (40 lx). The dark compartment served as a goal box and was connected to the light compartment via a u-shaped channel (4 cm × 4 cm × 8 cm) and a rectangular shaped doorway (4 cm × 4 cm). Each mouse conducted three consecutive trials, in which it was initially placed in the light compartment facing the channel and the latency to reach the goal box was measured. While in the first and second trial the channel provided free entrance into the goal box, in the third trial this channel was filled with 100 ml bedding material to create an obstruction for entering the goal box. Therefore, the latency to reach the goal box in the third trial represents not just learning, but also the problem-solving ability of an individual by means of overcoming an obstacle it has not encountered before. Between all trials the used bedding material was removed and the apparatus cleaned thoroughly with 70% ethanol. The parameters taken into account were the change in latency from the first to the second trial and the latency to reach the goal box in the third trial.

#### Faecal samples

To determine stress hormone and testosterone levels non-invasively, faecal corticosterone (FCMs) and testosterone metabolites (FTMs) were measured. Therefore, on PND 87 mice were transferred for a duration of 3 h (1 p.m.–4 p.m.) into new Makrolon type III cages to collect faecal samples. These collecting cages were equipped with a thin layer of bedding material and new enrichment (MouseHouse, tissue paper and a wooden stick). After the 3 h collecting phase, all faecal boli defecated were sampled and frozen at − 20 °C. Samples were dried and homogenised and an aliquot of 0.05 g each was extracted with 1 ml of 80% methanol. Finally, a 5α-pregnane-3β,11β,21-triol-20-one enzyme immunoassay to determine FCMs and a testosterone enzyme immunoassay to determine FTMs was used. Both were established and successfully validated to measure FCMs and FTMs in mice, respectively (for details see Refs.^65–67^). Intra- and inter-assay coefficients of variation were all below 10%.

#### Sucrose Consumption test

The SC is commonly used to investigate anhedonia in rodents^68^. Therefore, the mice’s preference for sweet, saccharated solutions in comparison to tap water was examined. In detail, the mice had for 72 h free access to two bottles, one containing tap water and the other one was filled with 3%—sucrose solution. Parameters measured were the total liquid and the relative amount of sucrose solution intake. Please note, due to technical reasons (i.e. spilled bottles) data points of 4 animals had to be excluded for this test.

#### Nest test

To assess the nest building ability of the animals, one hour prior to the onset of the dark phase, the shelter and tissue paper were removed from the cages for 24 h and a cotton nestlet (NES3600, Ancare) was provided. The quality of the nests was scored independently by two experimenters after 5 and 24 h. The definition of scores was adopted from Deacon^69^ and ranged from 1 to 5. For each time point the assigned scores of the two experimenters were averaged.

#### Home cage behaviour

Spontaneous (i.e. undisturbed) home cage behaviour was recorded on six days (PND 73 ± 1, PND 80 ± 1, PND 81 ± 1, PND 86, PND 92 and PND 93 ± 1) during the experimental test phase in the housing room. Observations were conducted during the active phase (9.15 a.m.–8.15 p.m.) under red light conditions by an experienced observer. On each of the 6 days, one observation session took place and lasted for 60 min. During each session, all mice of one experiment/mini-experiment were observed consecutively. The six observation sessions were conducted at different times of the day. They were evenly distributed across the active phase to enhance the generalisability of the observed behaviour. The behaviour of each mouse was recorded by instantaneous, focal animal sampling at intervals of 6 min, i.e. 10 times per session, thus resulting in 60 observations per mouse in total. The observed outcome measures were the relative amount of active observations (i.e. activity level) and the percentage of active observations, in which an animal was either observed ‘climbing’ (i.e. locomotion) or ‘drinking/feeding’ (i.e. maintenance behaviour). For definitions of observed behaviours see Supplementary Table S8. Please note, due to technical reasons one observation session of one mouse had to be excluded, resulting in 50 instead of 60 observations for this animal in total.

### Statistics

The analyses described in the following were conducted using 16 out of 23 outcome measures derived from 10 experimental test procedures. This selection was necessary to avoid any dependencies between several outcome measures derived from one test procedure. The selected 16 outcome measures (see Supplementary Tables S2–S7) had a correlation coefficient < 0.5 and were therefore assumed to be independent (cf.^49^). The whole selection process was completed by an experimenter blind to the specific outcome measures so that any biases in the selection process could be avoided. Outcome measures for exclusion were determined in a way that as few outcome measures as possible had to be excluded to warrant non-dependency. Whenever only two outcome measures were correlated with each other, it was randomly chosen which one was excluded. The whole selection process was done before the analyses I and II (see below) were conducted.

For the main analysis, the reproducibility of the strain comparisons was assessed and compared between both experimental designs using the following two approaches (I) Consistency of the strain effect across replicate experiments and (II) Estimation of how often and how accurately the replicate experiments predict the overall effect.

### (I) Consistency of the strain effect across replicate experiments

To assess the ‘strain-by-replicate experiment’-interaction as a measurement for reproducibility, the following linear mixed model (LMM, Eqs. 1a and 1b) was applied to both designs (conventional and mini-experiment).

1a $$y_{ijmk} =_{{}} \mu + a_{i} + b_{j} + {\text{ c}}_{{{\text{ij}}}} + d_{m} + f_{im} + \varepsilon_{ijmk} ,$$

where *i* = 1, …, *n*_*S*_, *j* = 1, …, *n*_*R*_, *m* = 1,…, *n*_*B*_ and *k* = 1,…, *n*_*ijm*_. *a*_*i*_ indicates the main effect of the *i*th level of strain (treatment); *b*_*j*_ represents replicate experiments as a random effect where *b*_*j*_ ~ N(0,σ_b_^2^); *c*_*ij*_ represents strain-by-replicate experiment-interaction as random effect where *c*_*ij*_ ~ N(0,σ_c_^2^); *d*_*m*_ represents block as a random effect where *d*_*m*_ ~ N(0,σ_d_^2^); *f*_*im*_ represents strain-by-block-interaction as a random effect where *f*_*im*_ ~ N(0,σ_f_^2^) and the error term *ε*_*ijmk*_ ∼ N(0,σ_e_^2^).

or written in layman terms:

1b $$y = {\text{`strain'}} + {\text{`replicate experiment'}} + {\text{`strain }} \times {\text{ replicate experiment'}}+ {\text{`block'}}+ {\text{`block}} \times {\text{strain'}},$$

where ‘strain’ was included as fixed factor and ‘replicate experiment’, ‘strain-by-replicate experiment’-interaction, ‘block’ and ‘block-by-strain’-interaction as random factors. The factor ‘block’ was included in accordance to the randomised block design used, in which mice sharing the same micro-environment were treated as one ‘block’ (i.e. same housing rack and testing time window, see Supplementary Fig. S1). In the mini-experiment design, each ‘block’ corresponded also to one mini-experiment. To meet the assumptions of parametric analysis, residuals were graphically examined for normal distribution, homoscedasticity, and the Shapiro–Wilk test was applied. When necessary, raw data were transformed using square root, inverse or logarithmic transformations (see Supplementary Tables S2–S7). Typically, the contribution of a fixed effect to a model is assessed by examine the F-values as they return the relative variance that is explained by the term against the total variance of the data. For a random effect, however, the F-values cannot be determined. Since the sample size in all replicate experiments was the same, the p-values of the ‘strain-by-replicate experiment’-interaction term were used as a proxy for the F-values. The p-values are a function of the chi-square value of a Likelihood Ratio test assessing the random effect and the degrees of freedom in the model. The degrees of freedom can be assumed to be the same in the analysis of both designs, since in both experimental designs the same sample size is used and the models have the same structure regarding the applied factor levels. Concerning the interaction term, higher p-values indicate more consistency of the strain effect across replicate experiments and thus better reproducibility. Subsequently, the p-values of the ‘strain-by-replicate experiment’-interaction term of all 16 outcome measures were compared between the conventional and mini-experiment design by using the Wilcoxon signed-rank test (paired, one-tailed) (statistical methodology adapted from the analysis of Ref.^32^).

### (II) Estimation of how often and how accurately the replicate experiments predict the overall effect

In the second analysis, the performance of each experimental design to predict the overall effect size was assessed by the coverage probability (Pc) and the proportion of accurate results (Pa). First, the overall effect size of each outcome measure and strain comparison were estimated by conducting a random-effect meta-analysis on the data of all replicate experiments independent of the experimental design. In detail, individual strain effect sizes and corresponding standard errors were calculated by applying the following linear mixed model (Eqs. 2a and 2b) to the data of each replicate experiment, separately.

2a $$y_{imk} = \mu + a_{i} + d_{m} + f_{im} + \varepsilon_{imk},$$

where *i* = 1, …, *n*_*S*_, *m* = 1,…, *n*_*B*_ and *k* = 1,…, *n*_*ijm*_. *a*_*i*_ indicates the main effect of the *i*th level of strain (treatment); *d*_*m*_ represents block as a random effect *d*_*m*_ ~ N(0,σ_d_^2^); *f*_*im*_ represents strain-by-block-interaction as a random effect *f*_*im*_ ~ N(0,σ_f_^2^) and the error term *ε*_*imk*_ ∼ N(0,σ_e_^2^).

or written in layman terms:

2b $$y= {\text{`strain'}} +{\text{`block'}}+ {\text{`block }} \times {\text{ strain'}},$$

where ‘strain’ was included as fixed effect and ‘block’ and the ‘block-by-strain’-interaction as random factors to account for the structure of the randomised block design in each replicate experiment (for details see section above and Supplementary Fig. S1).

The random-effect meta-analysis was based on the individual strain effect sizes and standard errors of all replicate experiments. It was conducted using the R-package ‘metafor’^70^ (Version 2.1.0) to return the overall effect sizes and corresponding CI_95_ of each outcome measure and strain comparison using following mixed effect model (Eqs. 3a and 3b).

3a $$S_{i} =_{{}} \mu + f_{i} + \varepsilon_{i} ,$$

where *i* = 1, …, *n*_*R*_. *S*_*i*_ represents the estimated strain effect sizes. *f*_*i*_ indicates replicate experiment as a random effect and the error term *ε*_*i*_ ∼ N(0,σ_e_^2^).

or written in layman terms:

3b $${\text{y}} = {\text{`replicate experiment'}}.$$

Following this step, individual mean strain differences and CI_95_ were computed based on the LMM in Eqs. (2a and 2b) using the R-package ‘lsmeans’^71^ (Version 2.30.0) for each replicate experiment of both designs, separately. In a next step, the Pc and Pa were assessed for each design. The Pc was calculated by counting how often the CI_95_ of the replicate experiments covered the overall effect size, whereas the Pa was determined by how often the replicate experiments in each design predicted the overall effect accurately concerning its statistical significance. For the latter, it was examined whether the CI_95_ of the overall pooled effect overlapped with 0 (i.e. overall not significant effect) or not (i.e. overall significant effect). In a next step, the Pa was calculated by counting all replicate experiments of each design that predicted the overall effect accurately. In detail, two requirements had to be met for a replicate experiment to be counted as predicting the overall effect accurately. The CI_95_ of the replicate experiment had to include the overall effect and if CI_95_ of the overall effect included 0, then the CI_95_ of the replicate experiment also had to include 0.

In the end, similar to the p-values of the interaction-term in the first analysis, the Pc and Pa ratios of all 16 outcome measures were compared between both designs by using the Wilcoxon signed-rank test (paired, one-tailed).

All statistical analyses were conducted and graphs created using the statistical software package ‘R’^72^, except for testing the correlation of outcome measures IBM SPSS Statistics (IBM Version 23) was used. Differences were considered to be significant for p ≤ 0.05.

## Supplementary information

Supplementary Information.

## Data Availability

The raw and processed data of the current study as well as the code for the analyses are available in the Figshare repositories. https://figshare.com/s/f4f219a35128dc70bb68 and https://figshare.com/s/afbc4523e12b58cd8140 and https://figshare.com/s/f164b25df58a2cb2dde4
