# Supplementary material for: Improving reproducibility in animal research by splitting the study population into several ‘mini-experiments’
Source: Sci Rep. 2020 Oct 6;10:16579. doi: 10.1038/s41598-020-73503-4 (PMC7538440; doi:10.1038/s41598-020-73503-4)
Supplement: Supplementary file 1 — Supplementary Information. [file 41598_2020_73503_MOESM1_ESM.docx]

**Improving reproducibility in animal research by splitting the study population into several ‘mini-experiments’**

*Vanessa Tabea von Kortzfleisch^a,b*^, Natasha A. Karp^c^, Rupert Palme^d^, Sylvia Kaiser^a,b^, Norbert Sachser^a,b^ & S. Helene Richter^a,b*^*

^a^ Department of Behavioural Biology, University of Münster

^b^ Otto Creutzfeldt Center for Cognitive and Behavioral Neuroscience, University of Münster

^c^ Data Sciences & Quantitative Biology, Discovery Sciences, R&D, AstraZeneca, Cambridge,

UK

^d^ Department of Biomedical Sciences, University of Veterinary Medicine, Vienna, Austria

Correspondence and requests for materials should be addressed to S.H.R. (email: [richterh@uni-muenster.de](mailto:richterh@uni-muenster.de)) or V.T.v.K. (email: [v_vonk01@uni-muenster.de](mailto:v_vonk01@uni-muenster.de))

**Supplementary Information**


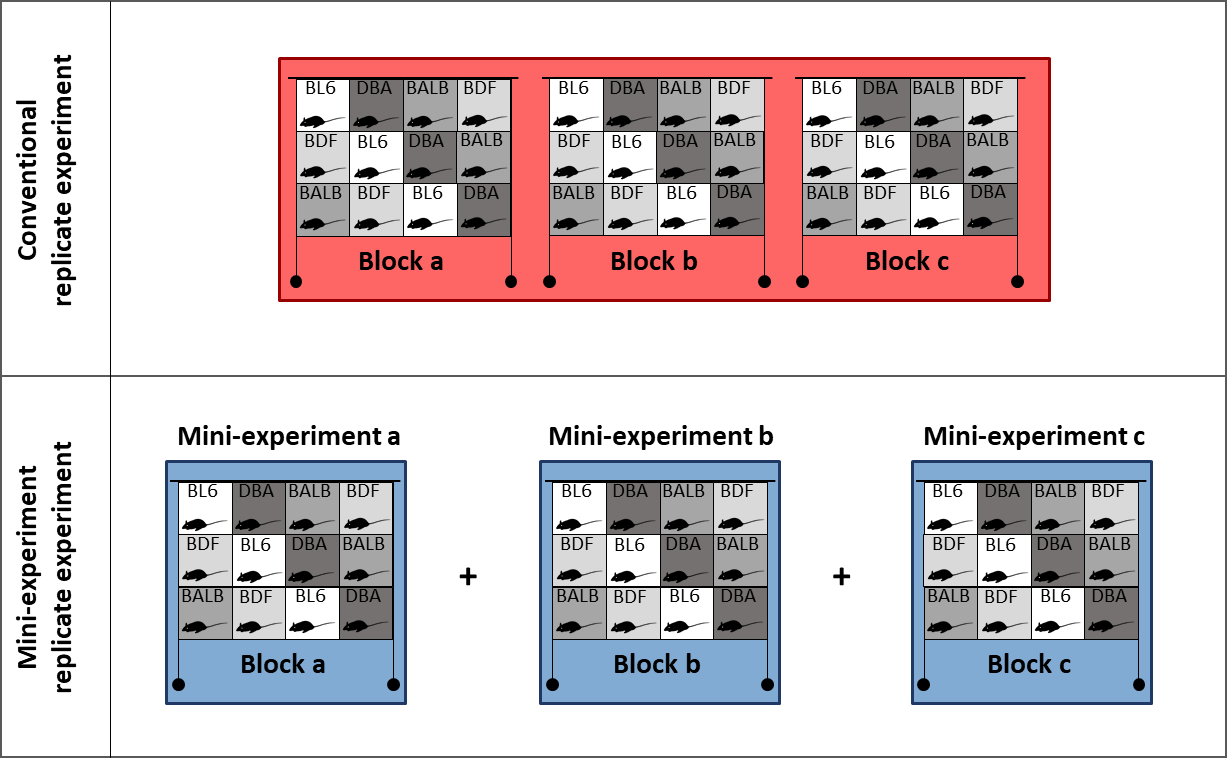


**Figure S1: Illustration of one exemplary conventional (red) and one exemplary mini-experiment (blue) replicate experiment according to the randomised block design used.** Regardless of the experimental design, each replicate experiment contained n = 9 mice per strain and was organised according to a randomised block design. In detail, always 3 mice of all four strains (12 in total) shared the following characteristics and were therefore treated as one ‘block’: First, they were housed in the same rack with the strains being allocated to their horizontal and vertical rack position in a balanced way. Furthermore, they were tested consecutively in the test procedures before another group of 12 mice were tested. Depending on the position of the specific rack in the housing room (e.g. disturbance, humidity, temperature, lighting) and the order of testing (e.g. testing time of the day, disturbance) environmental conditions may have differed between ‘blocks’. In the conventional design, all three ‘blocks’ of one replicate experiment were tested as one big experiment run simultaneously in time, whereas in the mini-experiment design, the ‘blocks’ were tested in independent mini-experiments spread over three time points a few weeks apart. Therefore, in the mini-experiment design, each ‘block’ corresponded also to one mini-experiment. With respect to the statistical analysis, the shared micro-environment in each ’block’ (e.g. same testing time window, same housing room and rack, possibly same mini-experiment) was included as a random factor in the linear mixed models applied to both designs. Thus, by using the same blocking structure in both designs, a comparable statistical analysis could be ensured. BL6: C57BL/6J strain, DBA: DBA/2N strain, BALB: BALB/cN strain, BDF: B6D2F1N strain

**Table S1**: Information about the date of testing and characteristics of the environmental background for each experiment in the conventional design (Con 1 - Con 4) and each mini-experiment in the mini-experiment design (Mini 1a – Mini 4c).


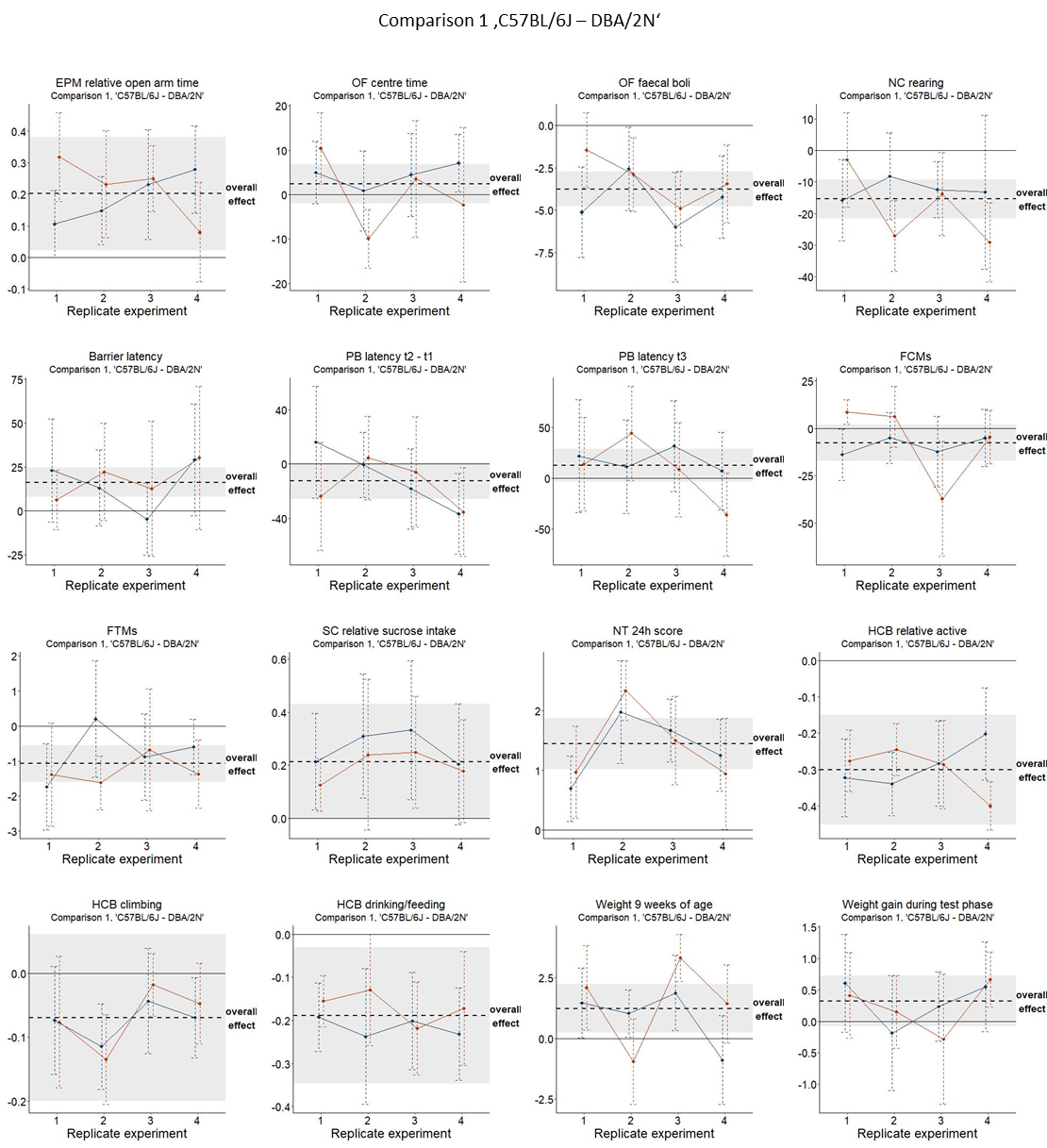


**Figure S2: Variation of mean strain differences across the four replicate experiments in the conventional (red) and the mini-experiments design (blue).** Shown are all 16 outcome measures for the strain comparison 1 ‘C57BL/6J – DBA/2N’. The black dashed line and the shaded area indicate the overall mean strain difference of this outcome measure and its corresponding 95% confidence interval (CI_95_). The black solid line reflects a null effect. Dots and vertical dashed lines reflect the mean strain differences and corresponding CI_95_ of the four replicate experiments in each design.


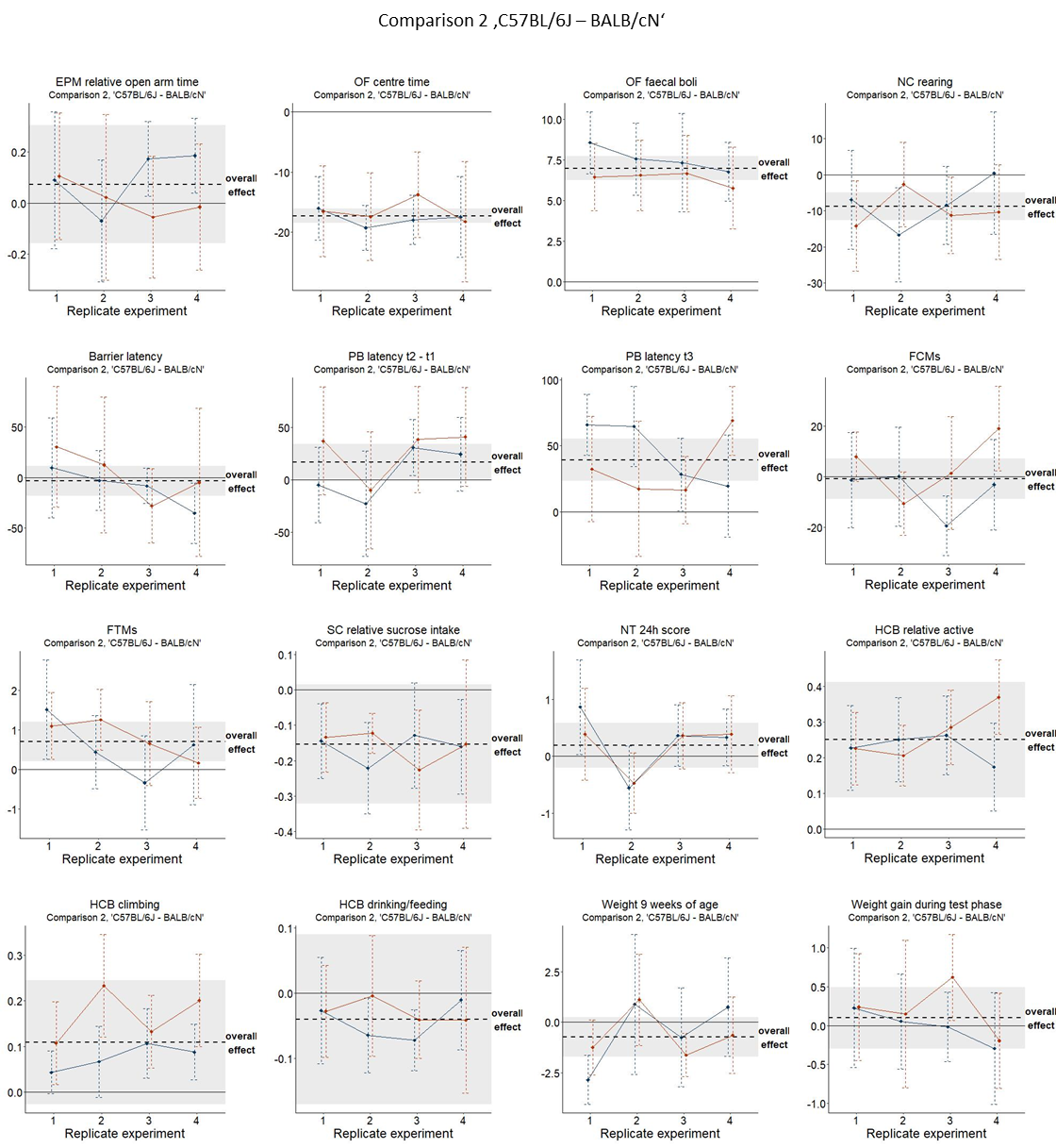


**Figure S3: Variation of mean strain differences across the four replicate experiments in the conventional (red) and the mini-experiments design (blue).** Shown are all 16 outcome measures for the strain comparison 2 ‘C57BL/6J – BALB/cN’. The black dashed line and the shaded area indicate the overall mean strain difference of this outcome measure and its corresponding 95% confidence interval (CI_95_). The black solid line reflects a null effect. Dots and vertical dashed lines reflect the mean strain differences and corresponding CI_95_ of the four replicate experiments in each design.


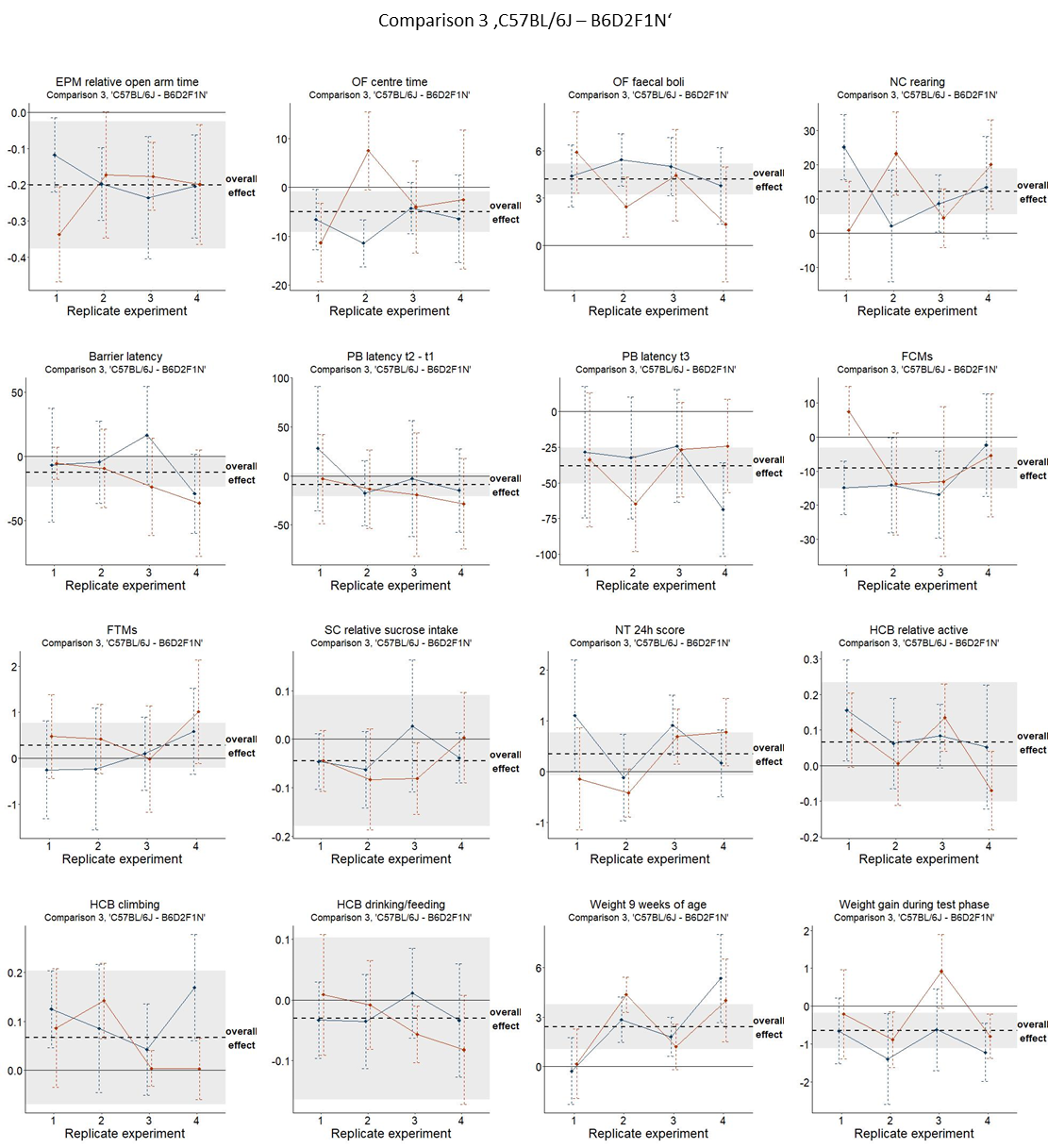


**Figure S4: Variation of mean strain differences across the four replicate experiments in the conventional (red) and the mini-experiments design (blue).** Shown are all 16 outcome measures for the strain comparison 3 ‘C57BL/6J – B6D2F1N’. The black dashed line and the shaded area indicate the overall mean strain difference of this outcome measure and its corresponding 95% confidence interval (CI_95_). The black solid line reflects a null effect. Dots and vertical dashed lines reflect the mean strain differences and corresponding CI_95_ of the four replicate experiments in each design.


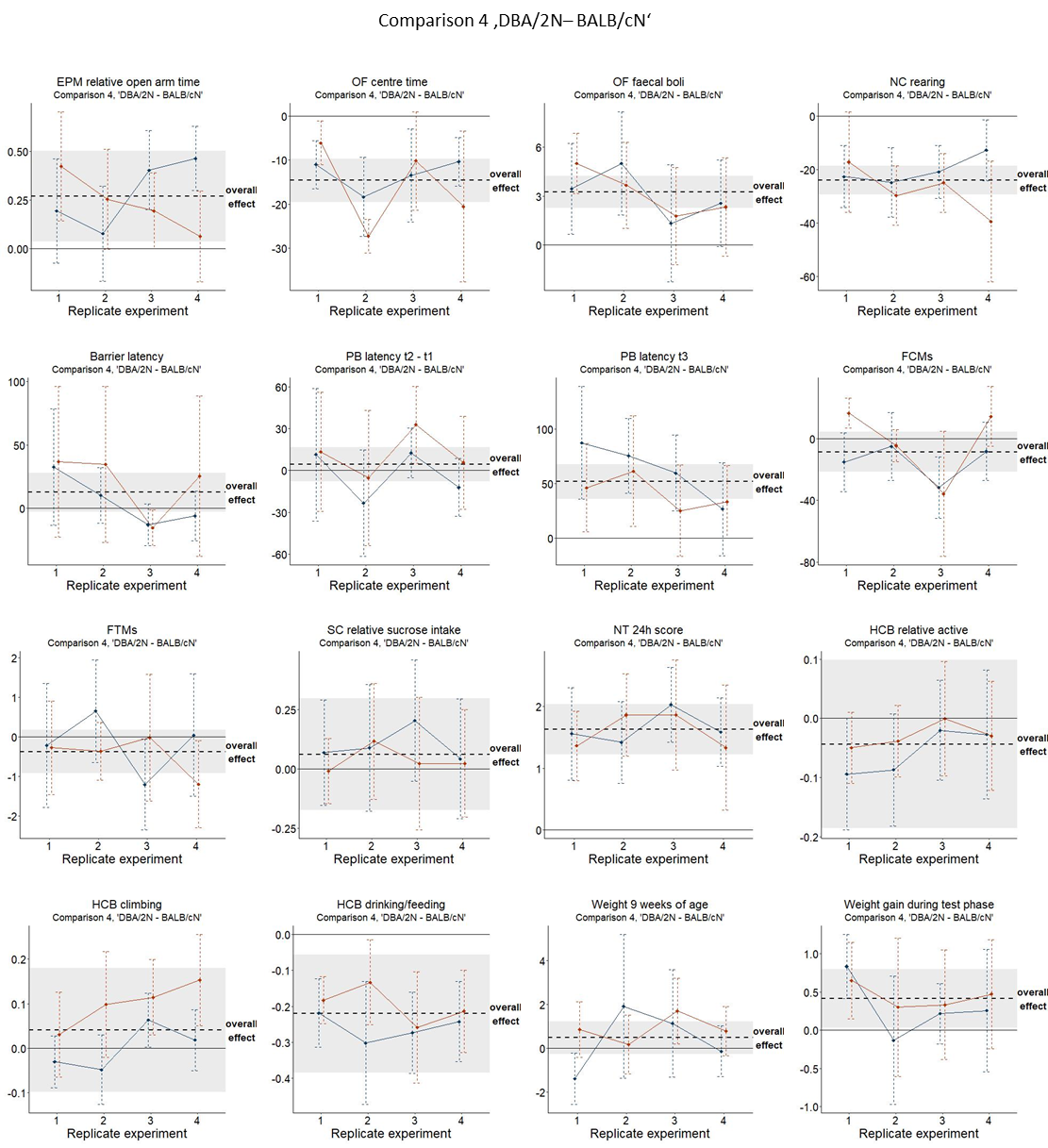


**Figure S5: Variation of mean strain differences across the four replicate experiments in the conventional (red) and the mini-experiments design (blue).** Shown are all 16 outcome measures for the strain comparison 4 ‘DBA/2N – BALB/cN’. The black dashed line and the shaded area indicate the overall mean strain difference of this outcome measure and its corresponding 95% confidence interval (CI_95_). The black solid line reflects a null effect. Dots and vertical dashed lines reflect the mean strain differences and corresponding CI_95_ of the four replicate experiments in each design.


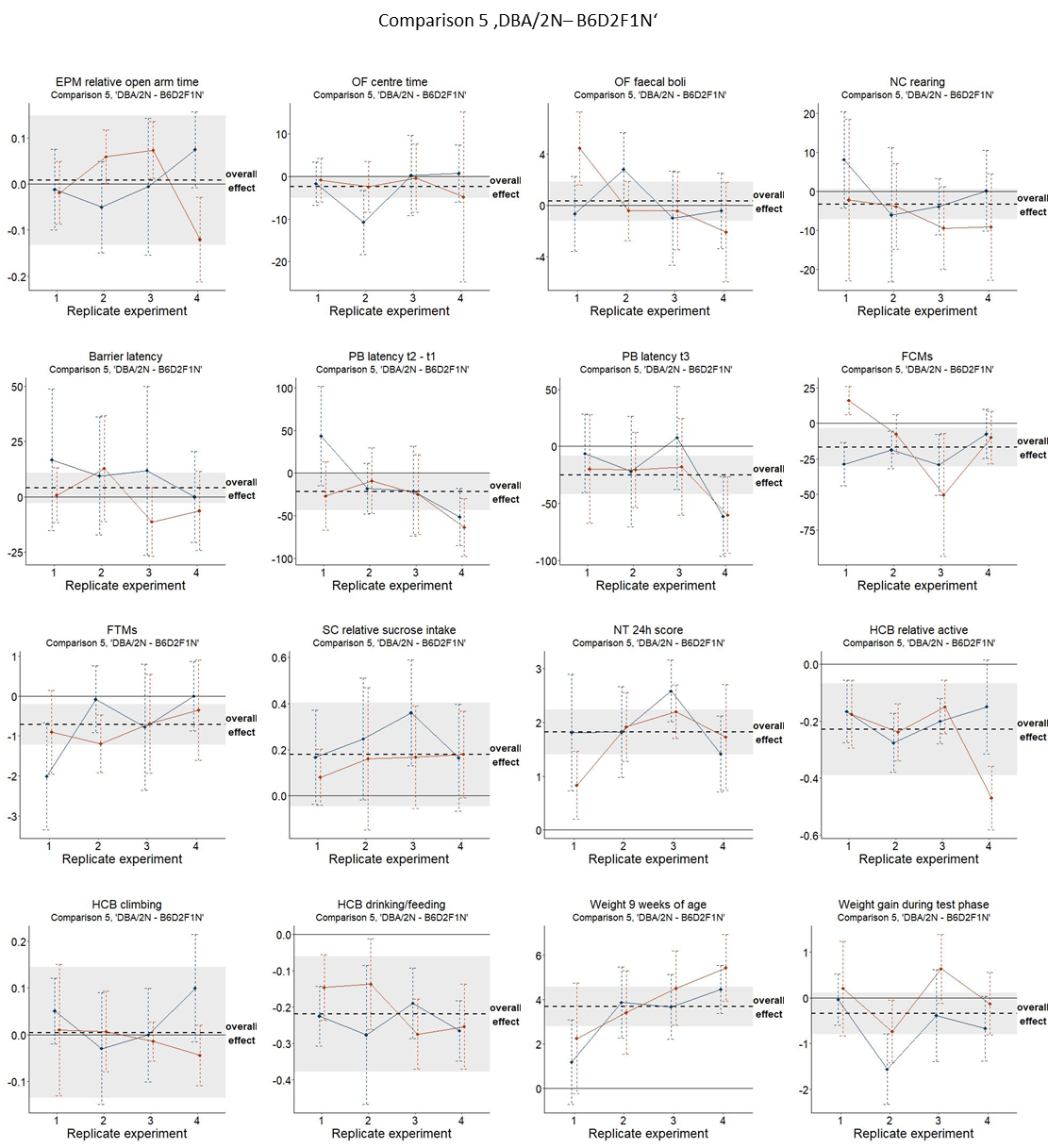


**Figure S6: Variation of mean strain differences across the four replicate experiments in the conventional (red) and the mini-experiments design (blue).** Shown are all 16 outcome measures for the strain comparison 5 ‘DBA/2N – B6D2F1N’. The black dashed line and the shaded area indicate the overall mean strain difference of this outcome measure and its corresponding 95% confidence interval (CI_95_). The black solid line reflects a null effect. Dots and vertical dashed lines reflect the mean strain differences and corresponding CI_95_ of the four replicate experiments in each design.


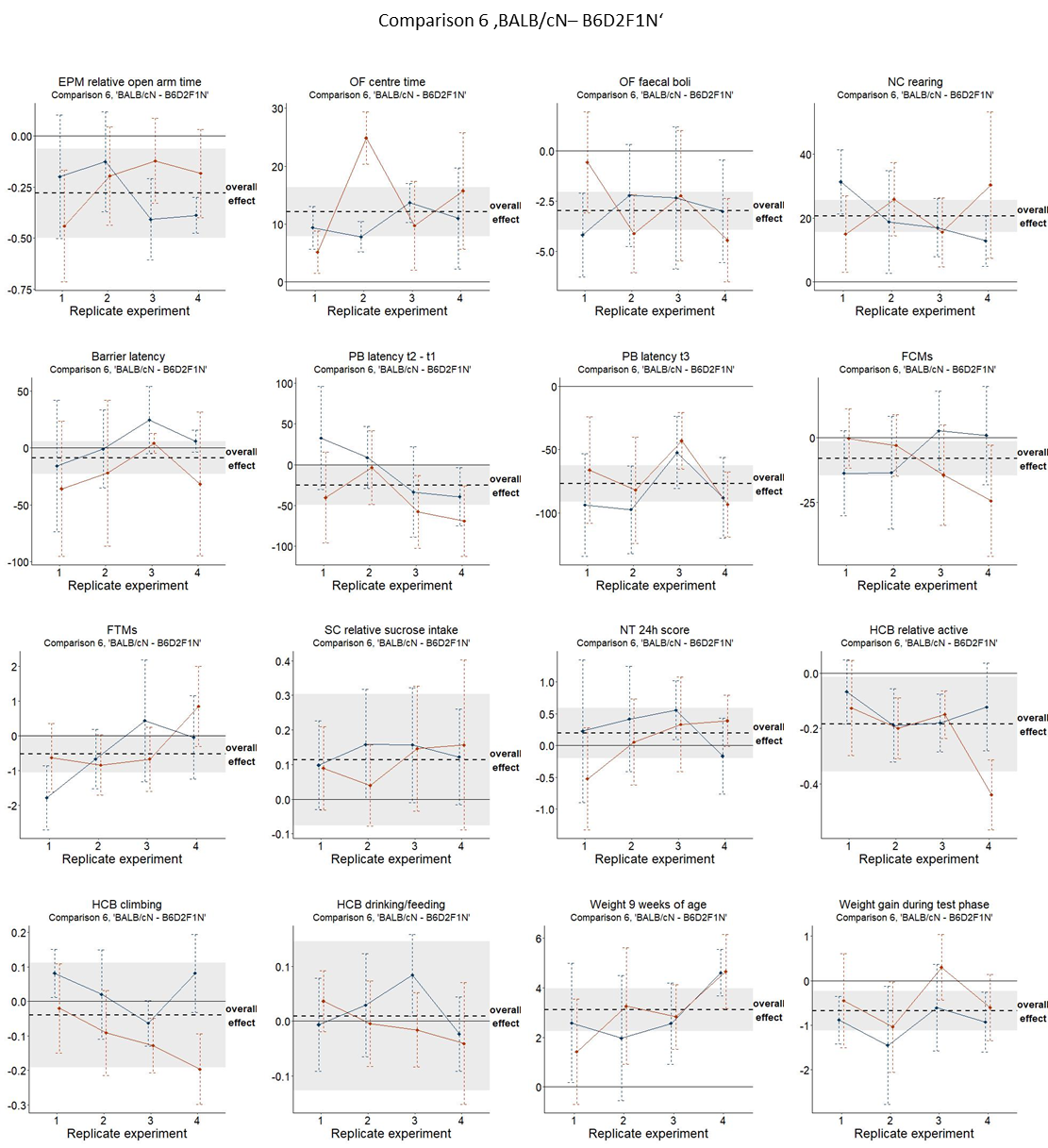


**Figure S7: Variation of mean strain differences across the four replicate experiments in the conventional (red) and the mini-experiments design (blue).** Shown are all 16 outcome measures for the strain comparison 6 ‘BALB/cN – B6D2F1N’. The black dashed line and the shaded area indicate the overall mean strain difference of this outcome measure and its corresponding 95% confidence interval (CI_95_). The black solid line reflects a null effect. Dots and vertical dashed lines reflect the mean strain differences and corresponding CI_95_ of the four replicate experiments in each design.

**Table S2**: Output of the linear mixed model (LMM) for both the conventional and mini-experiment design examining the strain comparison 1 ‘C57BL/6J – DBA/2N’. Presented are χ^2^- and p-values for the main effect of strain and the strain-by-replicate experiment interaction for all 16 selected outcome measures. Italic p-values represent significant main effects of the strain. Bold p-values indicate significant strain-by-replicate experiment interactions, highlighting impaired reproducibility in this experimental design and outcome measure. Transformations: log = log_10_ (y-1), sqrt = square root.


**Table S3**: Output of the linear mixed model (LMM) for both the conventional and mini-experiment design examining the strain comparison 2 ‘C57BL/6J – BALB/cN’. Presented are χ^2^- and p-values for the main effect of strain and the strain-by-replicate experiment interaction for all 16 selected outcome measures. Italic p-values represent significant main effects of the strain. Transformations: log = log_10_ (y-1), sqrt = square root, inverse = 1/(y-1).

**Table S4**: Output of the linear mixed model (LMM) for both the conventional and mini-experiment design examining the strain comparison 3 ‘C57BL/6J – B6D2F1N’. Presented are χ^2^- and p-values for the main effect of strain and the strain-by-replicate experiment interaction for all 16 selected outcome measures. Italic p-values represent significant main effects of the strain. Transformations: log = log_10_ (y-1), sqrt = square root, inverse = 1/(y-1).

**Table S5**: Output of the linear mixed model (LMM) for both the conventional and mini-experiment design examining the strain comparison 4 ‘DBA/2N – BALB/cN’. Presented are χ^2^- and p-values for the main effect of strain and the strain-by-replicate experiment interaction for all 16 selected outcome measures. Italic p-values represent significant main effects of the strain. Bold p-values indicate significant strain-by-replicate experiment interactions, highlighting impaired reproducibility in this experimental design and outcome measure. Transformations: log = log_10_ (y-1), sqrt = square root, inverse = 1/(y-1).

**Table S6**: Output of the linear mixed model (LMM) for both the conventional and mini-experiment design examining the strain comparison 5 ‘DBA/2N – B6D2F1N’. Presented are χ^2^- and p-values for the main effect of strain and the strain-by-replicate experiment interaction for all 16 selected outcome measures. Italic p-values represent significant main effects of the strain. Bold p-values indicate significant strain-by-replicate experiment interactions, highlighting impaired reproducibility in this experimental design and outcome measure. Transformations: log = log_10_ (y-1), sqrt = square root.

**Table S7**: Output of the linear mixed model (LMM) for both the conventional and mini-experiment design examining the strain comparison 6 ‘BALB/cN – B6D2F1N’. Presented are χ^2^- and p-values for the main effect of strain and the strain-by-replicate experiment interaction for all 16 selected outcome measures. Italic p-values represent significant main effects of the strain. Bold p-values indicate significant strain-by-replicate experiment interactions, highlighting impaired reproducibility in this experimental design and outcome measure. Transformations: log = log_10_ (y-1), sqrt = square root, inverse = 1/(y-1).


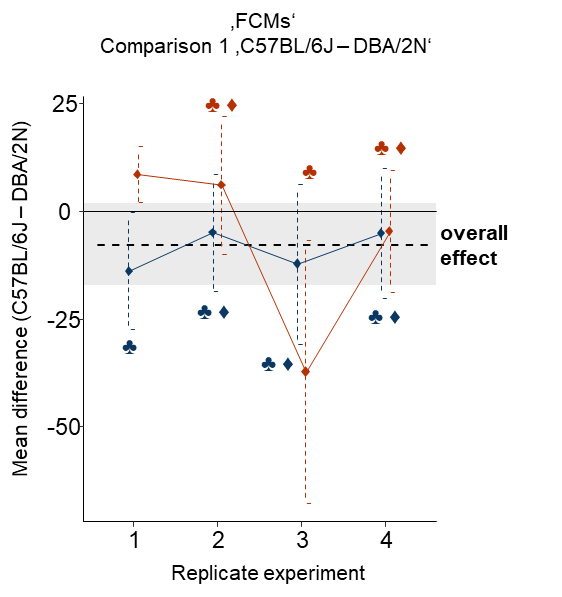


**Figure S8: Illustration of the assessment of the Pc (coverage probability) and Pa ratio (proportion of accurate results) at the example of one outcome measure.** Shown are mean strain differences in faecal corticosterone metabolite concentrations (FCMs) in comparison 1 ‘C57BL/6J – DBA/2N’ across the four replicate experiments in the conventional (red) and the mini-experiments design (blue). The black dashed line and the shaded area indicate the overall mean strain difference of this outcome measure and its corresponding 95% confidence interval (CI_95_). The black solid line reflects a null effect. Dots and vertical dashed lines reflect the mean strain differences and corresponding CI_95_ of the four replicate experiments in each design. Clubs and diamonds symbolise replicate experiments which cover the overall effect (♣, Coverage probability (Pc)) and predict it accurately (♦, Proportion of accurate results (Pa)), respectively. In detail, the Pc ratio reflects how often the CI_95_ of the replicate experiments included the overall effect size in contrast to the total amount of replicate experiments that were conducted. In the illustrate example, the conventional design is characterised by a Pc ratio of 0.75, whereas the mini-experiment design is characterised by a Pc ratio of 1. Concerning the Pa ratio, two requirements had to be met for a replicate experiment to be counted as predicting the overall effect accurately. The CI_95_ of the replicate experiment had to include the overall effect and if the CI_95_ of the overall effect included 0, then the CI_95_ of the replicate experiment also had to include 0. Thus, for the exemplarily shown outcome measure, the Pa ratio is 0.5 for the conventional design and 0.75 for the mini-experiment design.

**Table S8:** Description of behaviour patterns in the observation of spontaneous home cage behaviour.

| **Behaviour** | **Definition** |
| --- | --- |
| Active: | The mouse moves in a not specific defined way or movement of the bedding or nesting material can be seen. |
| Drinking/feeding | The mouse touches the nozzle of the water bottle or a food pellet with its snout or at least one front paw. |
| Climbing | The mouse touches the cage lid with at least one paw. At the same time no other paw is located on the ground. |
